# Supplementary material for: No support for white matter connectivity differences in the combined and inattentive ADHD presentations
Source: PLoS One. 2021 May 5;16(5):e0245028. doi: 10.1371/journal.pone.0245028 (PMC8099057; doi:10.1371/journal.pone.0245028)
Supplement: S1 Table — (DOCX) [file pone.0245028.s001.docx]

S1 Table. Correlations between the global network measures and the ADHD RS-IV item scores.

| ADHD combined and Inattentive Type Participants (*n* = 38) | | | |
| --- | --- | --- | --- |
|  | *r^2^* | *p* | *q* |
| ADHD-RS Sum of Items 1 - 9 |  |  |  |
| AUC_Mean Clustering coefficient | -.30 | .073 | .328 |
| AUC_Characteristic path length | -.02 | .898 | .898 |
| ADHD- RS Sum of Items 10 - 18 |  |  |  |
| AUC_ Mean Clustering coefficient | .10 | .547 | .777 |
| AUC_ Characteristic path length | .18 | .302 | .777 |
| Total Item Score |  |  |  |
| AUC_ Mean Clustering coefficient | .07 | .691 | .777 |
| AUC_ Characteristic path length | .14 | .412 | .777 |

AUC, area under the curve; ADHD-RS IV, attention deficit hyperactivity disorder rating scales - version 4; ADHD-RS Sum of Items 1 -9, inattention subscale; ADHD- RS Sum of Items 10 – 18, hyperactivity-impulsivity subscale; Total item score, the sum of 18 items involving the inattention and hyperactivity-impulsivity subscale.
