# Supplementary material for: No support for white matter connectivity differences in the combined and inattentive ADHD presentations
Source: PLoS One. 2021 May 5;16(5):e0245028. doi: 10.1371/journal.pone.0245028 (PMC8099057; doi:10.1371/journal.pone.0245028)
Supplement: S2 Table — (DOCX) [file pone.0245028.s002.docx]

S2 Table. Correlations between averaged FA values of the 46 white matter tracts and the ADHD-RS IV scores

| ADHD Combined and Inattentive Type Participants (*n* = 38) |  | | |  | |  |
| --- | --- | --- | --- | --- | --- | --- |
|  | | | *r^2^* | *p* | | *q* |
| ADHD-RS Sum of items 1 - 9 | | | |  | |  |
| Middle cerebellar peduncle | | -.090 | | | .595 | .998 |
| Pontine crossing tract | | .000 | | | .983 | .998 |
| Genu of corpus callosum | | -.250 | | | .143 | .998 |
| Body of corpus callosum | | -.190 | | | .282 | .998 |
| Splenium of corpus callosum | | .140 | | | .434 | .998 |
| Fornix (column and body of fornix) | | .010 | | | .945 | .998 |
| Corticospinal tract_Right | | .040 | | | .841 | .998 |
| Corticospinal tract_Left | | .050 | | | .777 | .998 |
| Medial lemniscus_Right | | -.020 | | | .903 | .998 |
| Medial lemniscus_Left | | -.070 | | | .681 | .998 |
| Inferior cerebellar peduncle_Right | | -.050 | | | .763 | .998 |
| Inferior cerebellar peduncle_Left | | -.050 | | | .777 | .998 |
| Superior cerebellar peduncle_Right | | -.040 | | | .836 | .998 |
| Superior cerebellar peduncle_Left | | .020 | | | .899 | .998 |
| Cerebral peduncle_Right | | .050 | | | .794 | .998 |
| Cerebral peduncle_Left | | -.030 | | | .850 | .998 |
| Anterior limb of internal capsule_Right | | -.200 | | | .238 | .998 |
| Anterior limb of internal capsule_Left | | -.350 | | | .039 | .998 |
| Posterior limb of internal capsule_Right | | .150 | | | .397 | .998 |
| Posterior limb of internal capsule_Left | | .080 | | | .663 | .998 |
| Retrolenticular part of internal capsule_Right | | .060 | | | .745 | .998 |
| Retrolenticular part of internal capsule_Left | | .040 | | | .832 | .998 |
| Anterior corona radiata_ Right | | -.270 | | | .121 | .998 |
| Anterior corona radiata_Left | | -.240 | | | .173 | .998 |
| Superior corona radiata_Right | | .080 | | | .667 | .998 |
| Superior corona radiata_Left | | .000 | | | .990 | .998 |
| Posterior corona radiata_Right | | -.030 | | | .845 | .998 |
| Posterior corona radiata_Left | | .020 | | | .895 | .998 |
| Posterior thalamic radiation_Right | | -.080 | | | .662 | .998 |
| Posterior thalamic radiation_Left | | .150 | | | .400 | .998 |
| Sagittal stratum_Right | | .010 | | | .958 | .998 |
| Sagittal stratum_Left | | .020 | | | .892 | .998 |
| External capsule_Right | | -.160 | | | .371 | .998 |
| External capsule_Left | | -.120 | | | .489 | .998 |
| Cingulum (cingulate gyrus)_Right | | -.150 | | | .403 | .998 |
| Cingulum (cingulate gyrus)_Left | | -.160 | | | .344 | .998 |
| Cingulum (hippocampus)_Right | | -.080 | | | .658 | .998 |
| Cingulum (hippocampus)_Left | | -.020 | | | .900 | .998 |
| Fornix/ Stria terminalis_Right | | .120 | | | .492 | .998 |
| Fornix/ Stria terminalis_Left | | .010 | | | .938 | .998 |
| Superior longitudinal fasciculus_Right | | -.020 | | | .892 | .998 |
| Superior longitudinal fasciculus_Left | | -.100 | | | .586 | .998 |
| Superior fronto-occipital fasciculus_Right | | .000 | | | .979 | .998 |
| Superior fronto-occipital fasciculus_Left | | -.070 | | | .671 | .998 |
| Uncinate fasciculus_Right | | .040 | | | .833 | .998 |
| Uncinate fasciculus_Left | | .130 | | | .472 | .998 |
| ADHD-RS Sum of items 10 - 18 | | | | |  |  |
| Middle cerebellar peduncle | | -.070 | | | .709 | .998 |
| Pontine crossing tract | | -.040 | | | .819 | .998 |
| Genu of corpus callosum | | .050 | | | .778 | .998 |
| Body of corpus callosum | | .140 | | | .438 | .998 |
| Splenium of corpus callosum | | .130 | | | .449 | .998 |
| Fornix (column and body of fornix) | | -.060 | | | .718 | .998 |
| Corticospinal tract_Right | | -.150 | | | .375 | .998 |
| Corticospinal tract_Left | | -.100 | | | .579 | .998 |
| Medial lemniscus_Right | | -.030 | | | .867 | .998 |
| Medial lemniscus_Left | | .040 | | | .798 | .998 |
| Inferior cerebellar peduncle_Right | | -.110 | | | .532 | .998 |
| Inferior cerebellar peduncle_Left | | -.060 | | | .724 | .998 |
| Superior cerebellar peduncle_Right | | -.360 | | | .032 | .998 |
| Superior cerebellar peduncle_Left | | -.200 | | | .246 | .998 |
| Cerebral peduncle_Right | | -.020 | | | .897 | .998 |
| Cerebral peduncle_Left | | -.060 | | | .725 | .998 |
| Anterior limb of internal capsule_Right | | .050 | | | .781 | .998 |
| Anterior limb of internal capsule_Left | | .080 | | | .662 | .998 |
| Posterior limb of internal capsule_Right | | .160 | | | .350 | .998 |
| Posterior limb of internal capsule_Left | | .260 | | | .133 | .998 |
| Retrolenticular part of internal capsule_Right | | -.040 | | | .810 | .998 |
| Retrolenticular part of internal capsule_Left | | .020 | | | .894 | .998 |
| Anterior corona radiata_ Right | | .040 | | | .823 | .998 |
| Anterior corona radiata_Left | | .060 | | | .736 | .998 |
| Superior corona radiata_Right | | .250 | | | .150 | .998 |
| Superior corona radiata_Left | | .180 | | | .306 | .998 |
| Posterior corona radiata_Right | | .240 | | | .164 | .998 |
| Posterior corona radiata_Left | | .300 | | | .077 | .998 |
| Posterior thalamic radiation_Right | | .000 | | | .998 | .998 |
| Posterior thalamic radiation_Left | | -.050 | | | .774 | .998 |
| Sagittal stratum_Right | | -.180 | | | .289 | .998 |
| Sagittal stratum_Left | | -.080 | | | .658 | .998 |
| External capsule_Right | | .170 | | | .342 | .998 |
| External capsule_Left | | .120 | | | .501 | .998 |
| Cingulum (cingulate gyrus)_Right | | .030 | | | .842 | .998 |
| Cingulum (cingulate gyrus)_Left | | .050 | | | .795 | .998 |
| Cingulum (hippocampus)_Right | | .010 | | | .933 | .998 |
| Cingulum (hippocampus)_Left | | .050 | | | .785 | .998 |
| Fornix/ Stria terminalis_Right | | -.010 | | | .953 | .998 |
| Fornix/ Stria terminalis_Left | | -.030 | | | .861 | .998 |
| Superior longitudinal fasciculus_Right | | -.010 | | | .949 | .998 |
| Superior longitudinal fasciculus_Left | | -.030 | | | .859 | .998 |
| Superior fronto-occipital fasciculus_Right | | .040 | | | .798 | .998 |
| Superior fronto-occipital fasciculus_Left | | -.010 | | | .938 | .998 |
| Uncinate fasciculus_Right | | .040 | | | .830 | .998 |
| Uncinate fasciculus_Left | | .260 | | | .127 | .998 |
| Total item score | |  | | |  |  |
| Middle cerebellar peduncle | | -.100 | | | .560 | .998 |
| Pontine crossing tract | | -.030 | | | .849 | .998 |
| Genu of corpus callosum | | -.080 | | | .656 | .998 |
| Body of corpus callosum | | .030 | | | .869 | .998 |
| Splenium of corpus callosum | | .180 | | | .297 | .998 |
| Fornix (column and body of fornix) | | -.050 | | | .777 | .998 |
| Corticospinal tract_Right | | -.120 | | | .497 | .998 |
| Corticospinal tract_Left | | -.060 | | | .727 | .998 |
| Medial lemniscus_Right | | -.040 | | | .837 | .998 |
| Medial lemniscus_Left | | .000 | | | .978 | .998 |
| Inferior cerebellar peduncle_Right | | -.120 | | | .488 | .998 |
| Inferior cerebellar peduncle_Left | | -.080 | | | .656 | .998 |
| Superior cerebellar peduncle_Right | | -.340 | | | .049 | .998 |
| Superior cerebellar peduncle_Left | | -.170 | | | .341 | .998 |
| Cerebral peduncle_Right | | .000 | | | .991 | .998 |
| Cerebral peduncle_Left | | -.070 | | | .689 | .998 |
| Anterior limb of internal capsule_Right | | -.060 | | | .751 | .998 |
| Anterior limb of internal capsule_Left | | -.100 | | | .565 | .998 |
| Posterior limb of internal capsule_Right | | .210 | | | .218 | .998 |
| Posterior limb of internal capsule_Left | | .260 | | | .126 | .998 |
| Retrolenticular part of internal capsule_Right | | -.010 | | | .957 | .998 |
| Retrolenticular part of internal capsule_Left | | .040 | | | .827 | .998 |
| Anterior corona radiata_ Right | | -.090 | | | .592 | .998 |
| Anterior corona radiata_Left | | -.060 | | | .727 | .998 |
| Superior corona radiata_Right | | .250 | | | .141 | .998 |
| Superior corona radiata_Left | | .160 | | | .373 | .998 |
| Posterior corona radiata_Right | | .190 | | | .264 | .998 |
| Posterior corona radiata_Left | | .280 | | | .108 | .998 |
| Posterior thalamic radiation_Right | | -.040 | | | .833 | .998 |
| Posterior thalamic radiation_Left | | .030 | | | .881 | .998 |
| Sagittal stratum_Right | | -.160 | | | .367 | .998 |
| Sagittal stratum_Left | | -.060 | | | .747 | .998 |
| External capsule_Right | | .070 | | | .688 | .998 |
| External capsule_Left | | .050 | | | .797 | .998 |
| Cingulum (cingulate gyrus)_Right | | -.040 | | | .823 | .998 |
| Cingulum (cingulate gyrus)_Left | | -.040 | | | .823 | .998 |
| Cingulum (hippocampus)_Right | | -.020 | | | .890 | .998 |
| Cingulum (hippocampus)_Left | | .030 | | | .859 | .998 |
| Fornix/ Stria terminalis_Right | | .050 | | | .782 | .998 |
| Fornix/ Stria terminalis_Left | | -.020 | | | .908 | .998 |
| Superior longitudinal fasciculus_Right | | -.020 | | | .904 | .998 |
| Superior longitudinal fasciculus_Left | | -.070 | | | .677 | .998 |
| Superior fronto-occipital fasciculus_Right | | .040 | | | .813 | .998 |
| Superior fronto-occipital fasciculus_Left | | -.050 | | | .786 | .998 |
| Uncinate fasciculus_Right | | .050 | | | .773 | .998 |
| Uncinate fasciculus_Left | | .290 | | | .090 | .998 |
