# Supplementary material for: No support for white matter connectivity differences in the combined and inattentive ADHD presentations
Source: PLoS One. 2021 May 5;16(5):e0245028. doi: 10.1371/journal.pone.0245028 (PMC8099057; doi:10.1371/journal.pone.0245028)
Supplement: S3 Table — (DOCX) [file pone.0245028.s003.docx]

S3 Table. Correlations between regional nodal degree of the 84 white matter regions and the ADHD-RS IV scores

| ADHD Combined and Inattentive Type Participants (*n* = 38) |  |  |  |
| --- | --- | --- | --- |
|  |  |  |  |
|  | *r^2^* | *p* | *q* |
| ADHD-RS Sum of items 1 - 9 |  |  |  |
| Superior temporal sulcus_Left | .040 | .802 | .965 |
| Caudal anterior cingulate_Left | -.170 | .321 | .761 |
| Caudal middle frontal_Left | -.070 | .679 | .930 |
| Corpus callosum_Left | .540 | .001 | .126 |
| Cuneus_Left | -.010 | .964 | .991 |
| Entorhinal_Left | -.140 | .395 | .775 |
| Fusiform_Left | -.370 | .025 | .385 |
| Inferior parietal_Left | .030 | .861 | .973 |
| Inferior temporal_Left | .250 | .131 | .635 |
| Isthmus cingulate_Left | -.140 | .400 | .775 |
| Lateral occipital_Left | .110 | .515 | .855 |
| Lateral orbitofrontal_Left | -.210 | .217 | .748 |
| Lingual_Left | -.270 | .105 | .630 |
| Medial orbitofrontal_Left | .240 | .150 | .668 |
| Middle temporal_Left | .060 | .743 | .955 |
| Parahippocampal_Left | -.340 | .042 | .433 |
| Paracentral_Left | .200 | .237 | .748 |
| Parsopercularis_Left | -.230 | .164 | .689 |
| Parsorbitalis_Left | -.010 | .945 | .991 |
| Parstriangularis_Left | .030 | .869 | .973 |
| Pericalcarine_Left | -.050 | .757 | .960 |
| Postcentral_Left | .160 | .338 | .762 |
| Posterior cingulate_Left | -.220 | .191 | .726 |
| Precentral_Left | .260 | .118 | .630 |
| Precuneus_Left | -.140 | .417 | .782 |
| Rostral anterior cingulate_Left | -.010 | .941 | .991 |
| Rostral middle frontal_Left | .350 | .031 | .411 |
| Superior frontal_Left | -.050 | .785 | .962 |
| Superior parietal_Left | .080 | .655 | .930 |
| Superior temporal_Left | -.170 | .308 | .756 |
| Supramarginal_Left | .040 | .823 | .973 |
| Frontalpole_Left | .050 | .762 | .960 |
| Temporalpole_Left | .160 | .358 | .762 |
| Transverse temporal_Left | -.370 | .026 | .385 |
| Insula_Left | -.020 | .885 | .973 |
| Superior temporal sulcus_Right | -.110 | .512 | .855 |
| Caudal anterior cingulate_Right | .000 | .979 | .991 |
| Caudal middle frontal_Right | .080 | .650 | .930 |
| Corpus callosum_Right | .060 | .718 | .947 |
| Cuneus_Right | -.210 | .210 | .748 |
| Entorhinal_Right | -.090 | .605 | .897 |
| Fusiform_Right | -.420 | .009 | .231 |
| Inferior parietal_Right | -.110 | .535 | .855 |
| Inferior temporal_Right | -.180 | .290 | .748 |
| Isthmus cingulate_Right | -.210 | .217 | .748 |
| Lateral occipital_Right | .010 | .935 | .991 |
| Lateral orbitofrontal_Right | -.340 | .041 | .433 |
| Lingual_Right | -.210 | .210 | .748 |
| Medial orbitofrontal_Right | -.040 | .804 | .965 |
| Middle temporal_Right | -.050 | .762 | .960 |
| Parahippocampal_Right | -.420 | .011 | .231 |
| Paracentral_Right | -.100 | .570 | .871 |
| Parsopercularis_Right | -.080 | .634 | .914 |
| Parsorbitalis_Right | .160 | .331 | .762 |
| Parstriangularis_Right | -.010 | .958 | .991 |
| Pericalcarine_Right | -.240 | .156 | .678 |
| Postcentral_Right | .170 | .306 | .756 |
| Posterior cingulate_Right | -.200 | .233 | .748 |
| Precentral_Right | .340 | .039 | .433 |
| Precuneus_Right | -.350 | .035 | .433 |
| Rostral anterior cingulate_Right | .070 | .666 | .930 |
| Rostral middle frontal_Right | .380 | .020 | .360 |
| Superior frontal_Right | .040 | .833 | .973 |
| Superior parietal_Right | .150 | .366 | .762 |
| Superior temporal_Right | .100 | .560 | .860 |
| Supramarginal_Right | .220 | .193 | .726 |
| Frontalpole_Right | .400 | .014 | .271 |
| Temporal pole_Right | .260 | .127 | .635 |
| Transverse temporal_Right | -.150 | .369 | .762 |
| Insula_Right | -.170 | .309 | .756 |
| Hippocampus_Left | .170 | .327 | .762 |
| Amygdala_Left | -.210 | .216 | .748 |
| Accumbens-area_Left | .170 | .312 | .756 |
| Caudate_Left | .200 | .226 | .748 |
| Putamen_Left | -.130 | .451 | .824 |
| Pallidum_Left | .220 | .193 | .726 |
| Thalamus-proper_Left | .110 | .534 | .855 |
| Hippocampus_Right | .140 | .397 | .775 |
| Amygdala_Right | -.180 | .276 | .748 |
| Accumbens-area_Right | .180 | .283 | .748 |
| Caudate_Right | .420 | .010 | .231 |
| Putamen_Right | -.010 | .975 | .991 |
| Pallidum_Right | .120 | .496 | .850 |
| Thalamus-proper_Right | -.260 | .123 | .633 |
| ADHD- RS Sum of Items 10 - 18 |  |  |  |
| Superior temporal sulcus_Left | -.190 | .261 | 0.748 |
| Caudal anterior cingulate_Left | .220 | .189 | 0.726 |
| Caudal middle frontal_Left | -.270 | .110 | 0.63 |
| Corpus callosum_Left | -.050 | .785 | 0.962 |
| Cuneus_Left | -.190 | .251 | 0.748 |
| Entorhinal_Left | .160 | .359 | 0.762 |
| Fusiform_Left | .140 | .419 | 0.782 |
| Inferior parietal_Left | -.230 | .180 | 0.72 |
| Inferior temporal_Left | -.030 | .846 | 0.973 |
| Isthmus cingulate_Left | .050 | .791 | 0.962 |
| Lateral occipital_Left | -.430 | .008 | 0.231 |
| Lateral orbitofrontal_Left | .160 | .343 | 0.762 |
| Lingual_Left | -.140 | .417 | 0.782 |
| Medial orbitofrontal_Left | .460 | .004 | 0.231 |
| Middle temporal_Left | -.360 | .029 | 0.406 |
| Parahippocampal_Left | .120 | .469 | 0.827 |
| Paracentral_Left | -.010 | .945 | 0.991 |
| Parsopercularis_Left | .150 | .389 | 0.775 |
| Parsorbitalis_Left | .300 | .071 | 0.559 |
| Parstriangularis_Left | .020 | .887 | 0.973 |
| Pericalcarine_Left | -.280 | .096 | 0.63 |
| Postcentral_Left | -.130 | .451 | 0.824 |
| Posterior cingulate_Left | .150 | .376 | 0.77 |
| Precentral_Left | .050 | .768 | 0.962 |
| Precuneus_Left | -.160 | .358 | 0.762 |
| Rostral anterior cingulate_Left | .200 | .227 | 0.748 |
| Rostral middle frontal_Left | -.300 | .074 | 0.565 |
| Superior frontal_Left | .110 | .520 | 0.855 |
| Superior parietal_Left | -.260 | .115 | 0.63 |
| Superior temporal_Left | -.040 | .794 | 0.962 |
| Supramarginal_Left | -.190 | .260 | 0.748 |
| Frontalpole_Left | .070 | .683 | 0.93 |
| Temporalpole_Left | .100 | .558 | 0.86 |
| Transverse temporal_Left | -.150 | .368 | 0.762 |
| Insula_Left | -.200 | .237 | 0.748 |
| Superior temporal sulcus_Right | -.060 | .709 | 0.94 |
| Caudal anterior cingulate_Right | .120 | .471 | 0.827 |
| Caudal middle frontal_Right | .070 | .662 | 0.93 |
| Corpus callosum_Right | -.030 | .859 | 0.973 |
| Cuneus_Right | -.050 | .754 | 0.96 |
| Entorhinal_Right | .160 | .359 | 0.762 |
| Fusiform_Right | .050 | .788 | 0.962 |
| Inferior parietal_Right | -.030 | .855 | 0.973 |
| Inferior temporal_Right | .070 | .701 | 0.935 |
| Isthmus cingulate_Right | -.190 | .257 | 0.748 |
| Lateral occipital_Right | .000 | .989 | 0.991 |
| Lateral orbitofrontal_Right | .180 | .296 | 0.753 |
| Lingual_Right | -.300 | .071 | 0.559 |
| Medial orbitofrontal_Right | .250 | .137 | 0.651 |
| Middle temporal_Right | -.100 | .559 | 0.86 |
| Parahippocampal_Right | -.010 | .944 | 0.991 |
| Paracentral_Right | -.040 | .794 | 0.962 |
| Parsopercularis_Right | .190 | .248 | 0.748 |
| Parsorbitalis_Right | .060 | .727 | 0.951 |
| Parstriangularis_Right | .040 | .834 | 0.973 |
| Pericalcarine_Right | -.180 | .283 | 0.748 |
| Postcentral_Right | -.100 | .546 | 0.855 |
| Posterior cingulate_Right | .300 | .069 | 0.559 |
| Precentral_Right | .150 | .381 | 0.774 |
| Precuneus_Right | -.270 | .110 | 0.63 |
| Rostral anterior cingulate_Right | -.010 | .970 | 0.991 |
| Rostral middle frontal_Right | -.010 | .931 | 0.991 |
| Superior frontal_Right | .060 | .736 | 0.951 |
| Superior parietal_Right | -.160 | .345 | 0.762 |
| Superior temporal_Right | -.050 | .773 | 0.962 |
| Supramarginal_Right | -.030 | .844 | 0.973 |
| Frontalpole_Right | .240 | .159 | 0.679 |
| Temporal pole_Right | .240 | .147 | 0.668 |
| Transverse temporal_Right | .080 | .635 | 0.914 |
| Insula_Right | -.120 | .467 | 0.827 |
| Hippocampus_left | -.120 | .476 | 0.827 |
| Amygdala_Left | .270 | .110 | 0.63 |
| Accumbens-area_Left | .120 | .470 | 0.827 |
| Caudate_Left | .260 | .114 | 0.63 |
| Putamen_Left | -.030 | .878 | 0.973 |
| Pallidum_Left | .420 | .009 | 0.231 |
| Thalamus-proper_Left | .290 | .085 | 0.63 |
| Hippocampus_Right | -.070 | .691 | 0.931 |
| Amygdala_Right | .230 | .168 | 0.694 |
| Accumbens-area_Right | .070 | .695 | 0.932 |
| Caudate_Right | .250 | .142 | 0.663 |
| Putamen_Right | -.120 | .486 | 0.839 |
| Pallidum_Right | .150 | .385 | 0.775 |
| Thalamus-proper_Right | .180 | .286 | 0.748 |
| Total Item Score |  |  |  |
| Superior temporal sulcus_Left | -.140 | .405 | 0.779 |
| Caudal anterior cingulate_Left | .100 | .545 | 0.855 |
| Caudal middle frontal_Left | -.270 | .110 | 0.63 |
| Corpus callosum_Left | .240 | .151 | 0.668 |
| Cuneus_Left | -.170 | .312 | 0.756 |
| Entorhinal_Left | .060 | .731 | 0.951 |
| Fusiform_Left | -.070 | .662 | 0.93 |
| Inferior parietal_Left | -.180 | .291 | 0.748 |
| Inferior temporal_Left | .100 | .541 | 0.855 |
| Isthmus cingulate_Left | -.040 | .834 | 0.973 |
| Lateral occipital_Left | -.310 | .061 | 0.559 |
| Lateral orbitofrontal_Left | .030 | .862 | 0.973 |
| Lingual_Left | -.260 | .120 | 0.63 |
| Medial orbitofrontal_Left | .520 | .001 | 0.126 |
| Middle temporal_Left | -.280 | .092 | 0.63 |
| Parahippocampal_Left | -.070 | .682 | 0.93 |
| Paracentral_Left | .090 | .581 | 0.882 |
| Parsopercularis_Left | .000 | .983 | 0.991 |
| Parsorbitalis_Left | .250 | .131 | 0.635 |
| Parstriangularis_Left | .040 | .834 | 0.973 |
| Pericalcarine_Left | -.270 | .110 | 0.63 |
| Postcentral_Left | -.030 | .881 | 0.973 |
| Posterior cingulate_Left | .010 | .933 | 0.991 |
| Precentral_Left | .180 | .287 | 0.748 |
| Precuneus_Left | -.210 | .222 | 0.748 |
| Rostral anterior cingulate_Left | .170 | .318 | 0.761 |
| Rostral middle frontal_Left | -.070 | .675 | 0.93 |
| Superior frontal_Left | .070 | .681 | 0.93 |
| Superior parietal_Left | -.190 | .266 | 0.748 |
| Superior temporal_Left | -.130 | .449 | 0.824 |
| Supramarginal_Left | -.140 | .396 | 0.775 |
| Frontalpole_Left | .090 | .610 | 0.899 |
| Temporalpole_Left | .170 | .323 | 0.761 |
| Transverse temporal_Left | -.320 | .051 | 0.494 |
| Insula_Left | -.180 | .274 | 0.748 |
| Superior temporal sulcus_Right | -.110 | .506 | 0.855 |
| Caudal anterior cingulate_Right | .100 | .544 | 0.855 |
| Caudal middle frontal_Right | .100 | .539 | 0.855 |
| Corpus callosum_Right | .010 | .972 | 0.991 |
| Cuneus_Right | -.160 | .356 | 0.762 |
| Entorhinal_Right | .090 | .605 | 0.897 |
| Fusiform_Right | -.180 | .283 | 0.748 |
| Inferior parietal_Right | -.080 | .630 | 0.914 |
| Inferior temporal_Right | -.040 | .827 | 0.973 |
| Isthmus cingulate_Right | -.270 | .102 | 0.63 |
| Lateral occipital_Right | .010 | .976 | 0.991 |
| Lateral orbitofrontal_Right | -.020 | .888 | 0.973 |
| Lingual_Right | -.370 | .025 | 0.385 |
| Medial orbitofrontal_Right | .190 | .253 | 0.748 |
| Middle temporal_Right | -.110 | .508 | 0.855 |
| Parahippocampal_Right | -.230 | .176 | 0.715 |
| Paracentral_Right | -.090 | .602 | 0.897 |
| Parsopercularis_Right | .130 | .459 | 0.827 |
| Parsorbitalis_Right | .140 | .419 | 0.782 |
| Parstriangularis_Right | .030 | .879 | 0.973 |
| Pericalcarine_Right | -.280 | .093 | 0.63 |
| Postcentral_Right | .000 | .991 | 0.991 |
| Posterior cingulate_Right | .160 | .359 | 0.762 |
| Precentral_Right | .310 | .066 | 0.559 |
| Precuneus_Right | -.410 | .011 | 0.231 |
| Rostral anterior cingulate_Right | .030 | .847 | 0.973 |
| Rostral middle frontal_Right | .190 | .271 | 0.748 |
| Superior frontal_Right | .070 | .689 | 0.931 |
| Superior parietal_Right | -.060 | .734 | 0.951 |
| Superior temporal_Right | .010 | .957 | 0.991 |
| Supramarginal_Right | .090 | .614 | 0.9 |
| Frontalpole_Right | .410 | .011 | 0.231 |
| Temporal pole_Right | .340 | .038 | 0.433 |
| Transverse temporal_Right | -.010 | .954 | 0.991 |
| Insula_Right | -.200 | .245 | 0.748 |
| Hippocampus_Left | -.020 | .917 | 0.991 |
| Amygdala_Left | .120 | .475 | 0.827 |
| Accumbens-area_Left | .190 | .248 | 0.748 |
| Caudate_Left | .330 | .043 | 0.433 |
| Putamen_Left | -.090 | .600 | 0.897 |
| Pallidum_Left | .480 | .003 | 0.231 |
| Thalamus-proper_Left | .300 | .069 | 0.559 |
| Hippocampus_Right | .020 | .922 | 0.991 |
| Amygdala_Right | .100 | .542 | 0.855 |
| Accumbens-area_Right | .150 | .369 | 0.762 |
| Caudate_Right | .430 | .008 | 0.231 |
| Putamen_Right | -.100 | .537 | 0.855 |
| Pallidum_Right | .190 | .268 | 0.748 |
| Thalamus-proper_Right | .020 | .905 | 0.987 |
